# Supplementary material for: Estimating effects of serum vitamin B12 levels on psychiatric disorders and cognitive impairment: a Mendelian randomization study
Source: Commun Med (Lond). 2025 Jul 30;5:316. doi: 10.1038/s43856-025-01043-x (PMC12311191; doi:10.1038/s43856-025-01043-x)
Supplement: Supplementary file 2 — Supplementary Information [file 43856_2025_1043_MOESM2_ESM.pdf]

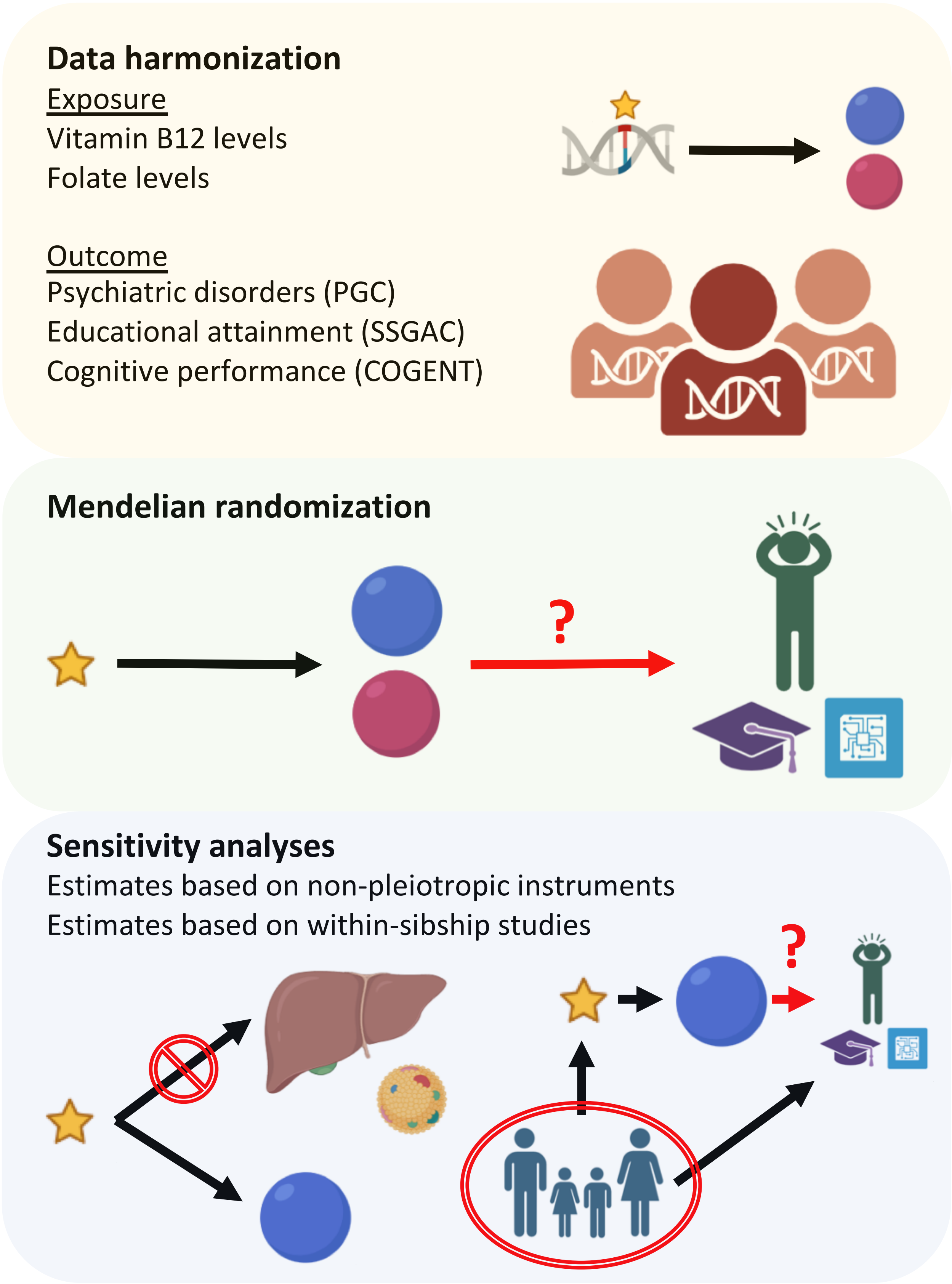

Supplementary Figure 1. Overview of study design. Summary statistics of large-scale genome-wide association studies were obtained for serum vitamin B12 and folate levels, eight psychiatric disorders, educational attainment, and cognitive performance. Mendelian randomization and multiple sensitivity analyses were performed to estimate the potential effects of vitamin B12 and folate levels. Within-sibship studies reduce confounding in population-based genome-wide association studies by controlling for demographic effects and indirect genetic effects.

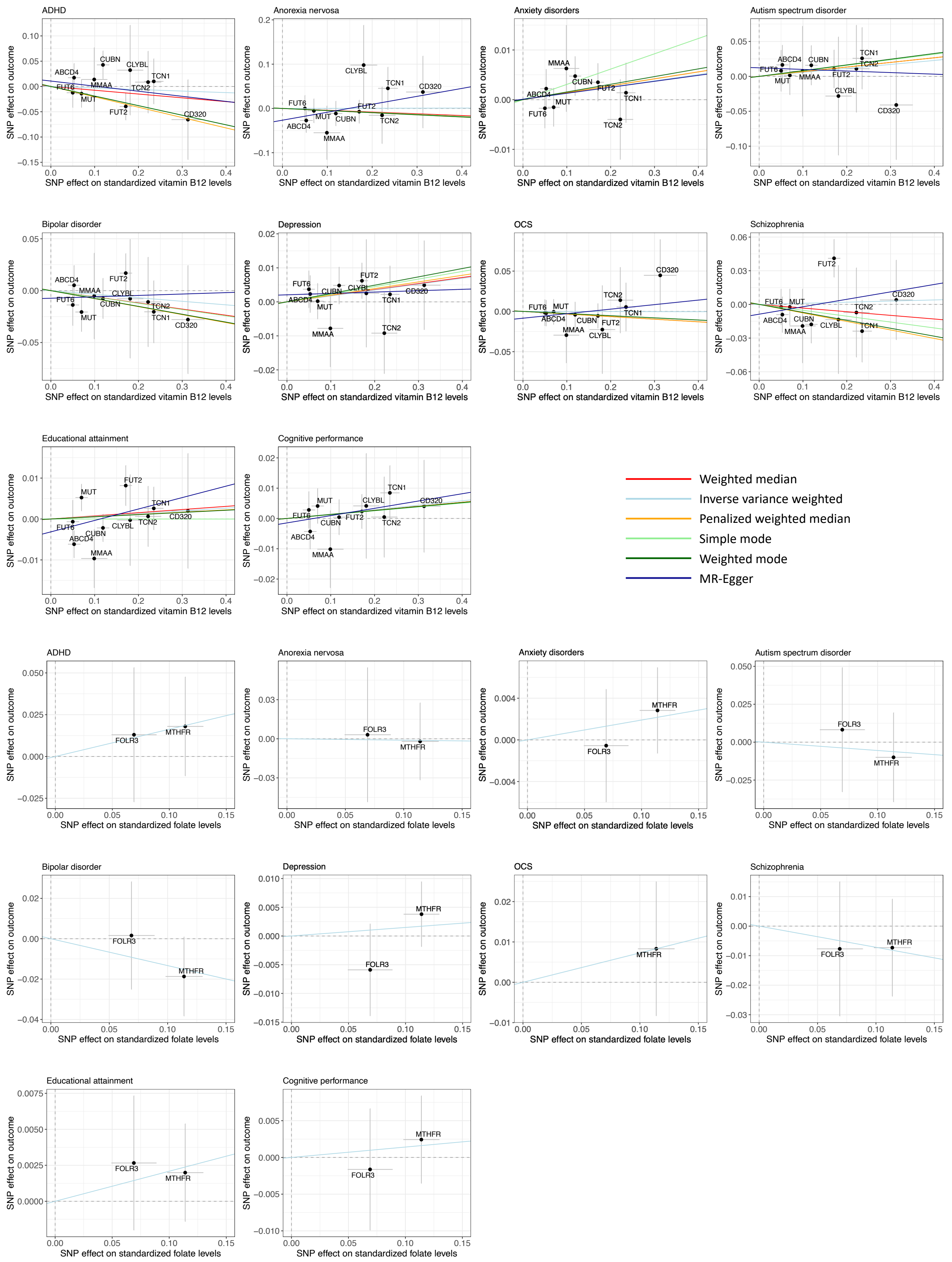

Supplementary Figure 2. Scatter plots comparing the genetic instrument-outcome and the genetic instrument-exposure associations. Primary results were obtained using the weighted median method and the inverse variance weighted regression method for vitamin B12 levels and folate levels, respectively. The mapped genes of the respective genetic instruments are indicated. The slopes of the colored lines represent estimates obtained through different Mendelian randomization methods. Error bars represent 95% confidence intervals.
