# Supplementary material for: Estimating effects of serum vitamin B12 levels on psychiatric disorders and cognitive impairment: a Mendelian randomization study
Source: Commun Med (Lond). 2025 Jul 30;5:316. doi: 10.1038/s43856-025-01043-x (PMC12311191; doi:10.1038/s43856-025-01043-x)
Supplement: Supplementary file 3 — Description of Additional Supplementary files [file 43856_2025_1043_MOESM3_ESM.pdf]

Description of Additional Supplementary files

File name: Supplementary Data 1

Description: Genetic instruments of vitamin B12 and folate levels.

File name: Supplementary Data 2

Description: Phenome-wide associations with genetic instruments.

File name: Supplementary Data 3

Description: Within-sibship genome wide association studies.

File name: Supplementary Data 4

Description: Estimated associations between vitamin B12 and folate levels and anemia based on exome-sequencing data from the UK Biobank.

File name: Supplementary Data 5

Description: Mendelian randomization results using vitamin B12 as exposure.

File name: Supplementary Data 6

Description: Mendelian randomization results using folate as exposure.

File name: Supplementary Data 7

Description: Sensitivity analyses after excluding FUT2 variant.

File name: Supplementary Data 8

Description: Sensitivity analyses after excluding all possibly pleiotropic variants.

File name: Supplementary Data 9

Description: Mendelian randomization results based on within-sibship genome-wide association studies of depressive symptoms, educational attainment, and cognitive performance
